# Combined Arthroscopic Medial Meniscal Root Repair and Centralization Using All‐Suture Anchors Without a Posteromedial Portal

**DOI:** 10.1002/atn2.70155

**Published:** 2026-05-24

**Authors:** Coşkun Genç, Burak Menderes Akdoğan, Ilias Fanourgiakis, Panagiotis Antzoulas, Fatih Barça, Halis Atıl Atilla

**Affiliations:** ^1^ Department of Orthopaedics and Traumatology Ankara Etlik City Hospital Ankara Turkey; ^2^ Department of Orthopaedics and Traumatology KAT Attica General Hospital Athens Greece; ^3^ Department of Orthopaedics and Traumatology General University Hospital of Patras Patras Greece

## Abstract

Medial meniscus posterior root tears frequently result in meniscal extrusion and disruption of normal tibiofemoral contact mechanics. Several techniques, including transtibial pullout and anchor‐based repair methods, are used for root repair and meniscus centralization. We describe a combined method of arthroscopic medial meniscal root repair and centralization using all suture anchors without a posteromedial portal. In this technical note, we provide a comprehensive overview of the above technique accompanied by a video demonstration.

## Graphical abstract

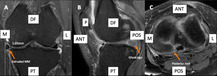

**VIDEO 1:** Arthroscopic demonstration of sequential meniscal centralization followed by posterior root repair for a medial meniscus posterior root tear in the left knee of a 51‐year‐old female patient, performed using two 3.0 mm all‐suture anchors without a posteromedial portal. The procedure is performed with the patient in the supine position. Key steps include arthroscopic portal placement, medial meniscus centralization, tibial tunnel creation, all‐suture anchor insertion, suture shuttling through the medial meniscus posterior root, knot tying, final reduction and fixation of the medial meniscus posterior root. Video content can be viewed at https://doi.org/10.1002/atn2.70155.

Meniscal root tears lead to loss of hoop tension, meniscal extrusion, and increased articular cartilage contact pressures.^1^, ^2^ Failure to recognize and repair medial meniscus posterior root tears may result in rapid progression of osteoarthritis in the medial compartment.^3^, ^4^ Moreover, meniscal extrusion has been shown to correlate with progressive joint instability and the onset of osteoarthritis.^5^, ^6^ Prior studies have shown that postoperative meniscal extrusion may persist despite successful root repair.^7^, ^8^ Meniscal centralization is indicated when extrusion exceeds 40% of the total meniscal width, as quantified by the Meniscal Extrusion Index.^9^ Early clinical results of centralization appear promising.^10^, ^11^

Several studies have reported favorable clinical and healing outcomes following medial meniscus posterior root tear repair using both anchor‐based techniques and the transtibial pull‐out method.^12^, ^13^, ^14^ Furthermore, several techniques including transtibial pullout and anchor‐based repair methods, are used for root repair and meniscus centralization.

In this Technical Note, root repair and meniscal centralization are performed using two 3.0 mm FiberWire‐equivalent (Seges, Ankara, Turkey) suture anchors. Unlike previously described anchor‐based root repair techniques, this method uses antegrade tibial drilling from the anteromedial tibial metaphysis for tunnel creation, followed by retrograde insertion and fixation of an all‐suture anchor within the tunnel. Centralization aims to re‐establish the native position of the medial meniscus by deploying all‐suture anchors along the medial border of the medial tibial plateau, with sutures passed through the meniscocapsular junction to achieve controlled medial meniscal centralization.

## SURGICAL TECHNIQUE

**FIGURE 1:**
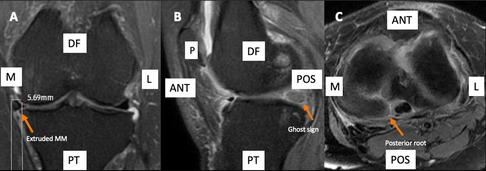
Magnetic resonance imaging findings of a medial meniscus posterior root tear shown on coronal (A), sagittal (B), and axial (C) images. (A) Coronal image showing the amount of medial meniscal extrusion, highlighted by the arrow. (B) Sagittal image illustrating the *ghost sign*, a pathognomonic finding indicating the absence of the medial meniscus posterior root, as indicated by the arrow. (C) Axial image showing increased vertical signal intensity at the posterior root region of the medial meniscus, with the arrow marking the affected area. (ANT, anterior; DF, distal femur; L, lateral; M, medial; P, patella; POS, posterior; PT, proximal tibia.)

A detailed evaluation of preoperative imaging is essential for surgical planning. Figure 1 shows a typical magnetic resonance imaging revealing a medial meniscus posterior root tear associated with meniscal extrusion. The equipment required for this procedure is listed in Table 1. Key steps are summarized in Table 2, and pearls and pitfalls are summarized in Table 3. The accompanying Video illustrates the performance of the technique described in detail below (Video 1).

**TABLE 1:** The Equipment Required for This Procedure

**Instrumentation**	**Centralization**
Arthroscopic shaver	3.0 mm all suture anchor (Seges, Ankara, Turkey)
Radiofrequency ablator	Suture Passer
**Posterior Root Repair**	No. 0 PDS
3.0 mm all suture anchor (Seges, Ankara, Turkey)	2.7 mm drill (Seges, Ankara, Turkey)
Meniscus Root Repair Aiming guide (Arthrex, Naples, FL)	
2.7 mm drill (Seges, Ankara, Turkey)	
No. 0 PDS	
Mini Scorpion Suture Passer (Arthrex, Naples, FL)	
Silicone cannula (Arthrex, Naples, FL)	

PDS, Polydioxanone Suture.

**TABLE 2:** Key Steps for This Procedure

**Key Steps**
Recognize tear pattern, confirm its reducibility using a suture retriever or a grasper
Use FM and AL portals and create an AM portal proximal and 2 cm medial to the standard FM portal
Prepare to improve exposure to the medial compartment through judicious percutaneous MCL posterior fibers pie‐crusting
First perform centralization, then the posterior root repair

AL, anterolateral; AM, accessory medial; FM, far medial; MCL, medial collateral ligament.

**TABLE 3:** Pearls and Pitfalls for This Procedure

**Pearls—Meniscus centralization**
Optimize proximal and medial position of the AM portal with consideration of the anchor insertion angle
Pass the suture passer precisely through the meniscocapsular junction
Before knot tying, to prevent any soft tissue interposition, withdraw the fiber pair once more extra‐articularly through the portal where the knot will be advanced
**Pearls—Meniscus root repair**
The suture limbs of the suture anchor intended for delivery, should be engaged by the PDS loop at their midpoint to ensure controlled passage
A suture retriever may be used to facilitate the placement of the all‐suture anchor within the tibial tunnel
Only one limb of the second suture pair was passed through the meniscus in order to increase the contact area between the root and footprint
**Pitfalls**
Insufficient mobilization of the meniscus before centralization
Inadequate anchor fixation in poor‐quality or osteoporotic tibial bone
Improper placement of the all‐suture anchor outside the native root footprint

AM, accessory medial; PDS, polydioxanone suture.

**FIGURE 2:**
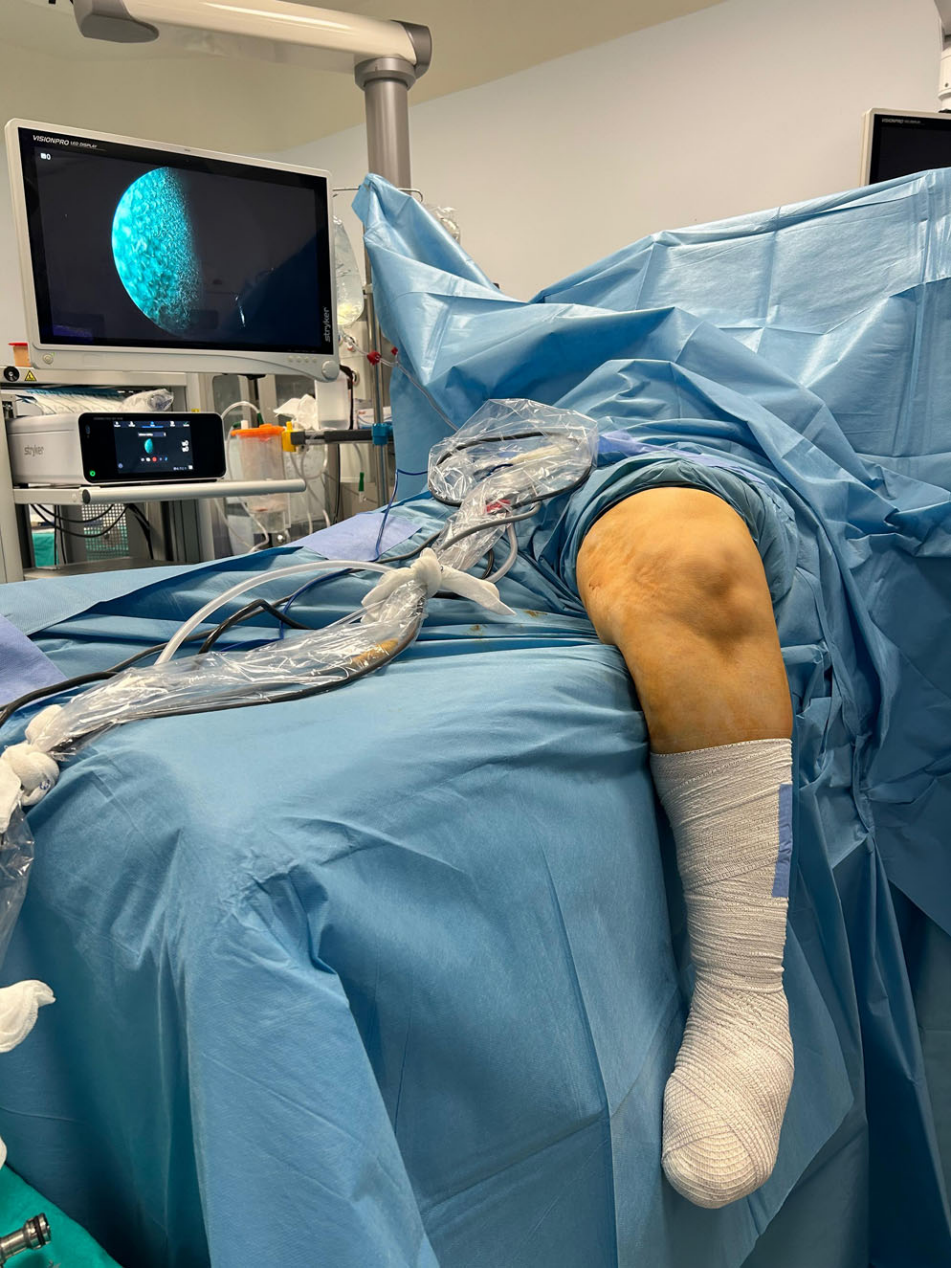
The patient is in the supine position with the left knee exposed and accessible. The operative left lower extremity is positioned to allow application of a valgus stress test without restricting the knee range of motion, with the leg allowed to hang freely. An anterior view of the knee is shown.

The operating table height is adjusted appropriately, and the patient is placed in the supine position. After the tourniquet is inflated, the operative extremity is allowed to hang freely over the edge of the table without restriction of the range of motion (Figure 2).

**FIGURE 3:**
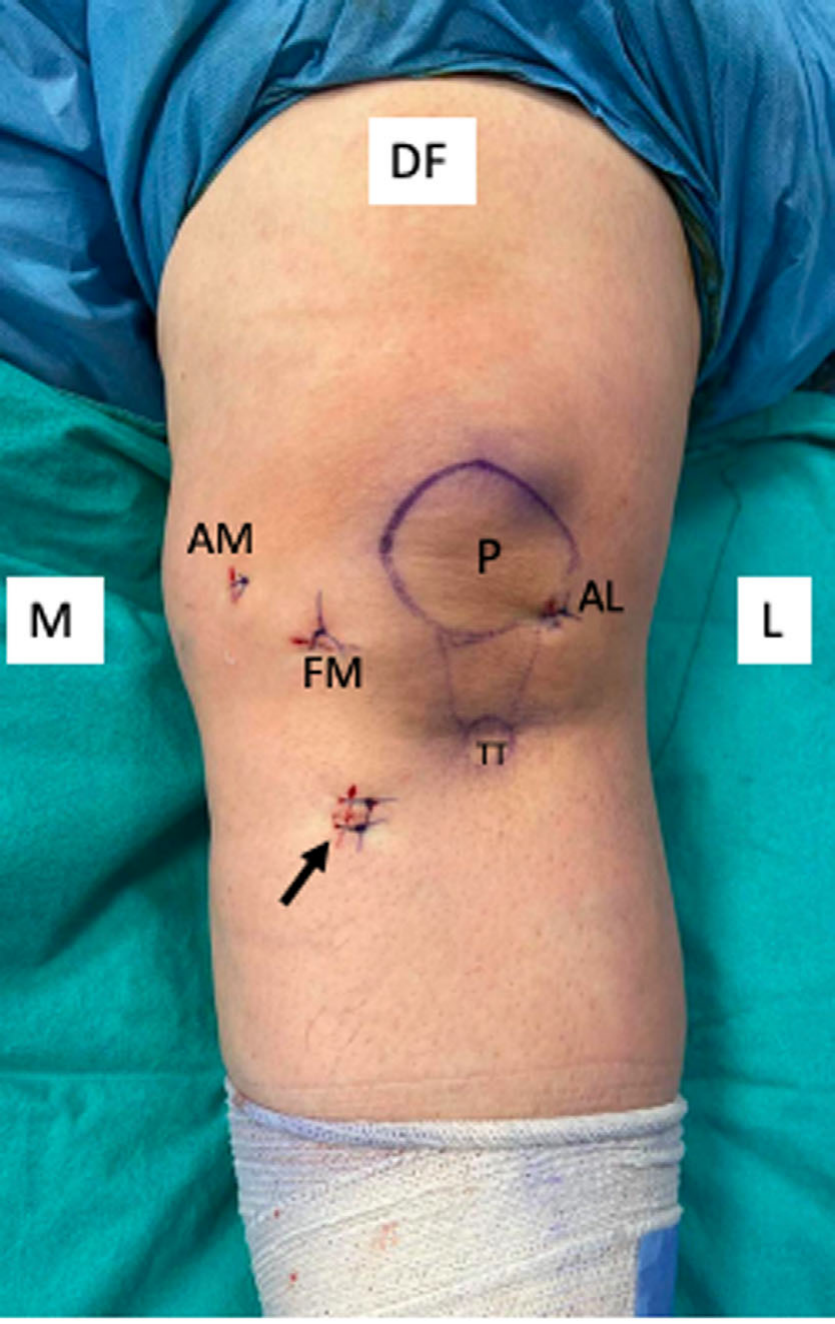
Anterior view of the left knee showing arthroscopic portal placement. The AL portal, used as the visualization portal, the FM portal, and the AM portal used for anchor insertion during meniscal centralization are marked on the skin. The arrow indicates a small incision created to allow the medial meniscus posterior root repair guide to be seated against the anterior cortex of the tibia for tibial tunnel creation. (AL, anterolateral; AM, accessory medial; DF, distal femur; FM, far medial; L, lateral; M, medial; P, patella; TT, tibial tubercle.)

Diagnostic arthroscopy is performed through the anterolateral (AL) and the far medial (FM) portals, which are created using a No.11 scalpel blade (Figure 3).

### Centralization

The procedure begins with percutaneous pie‐crusting of the posterior fibers of the superficial medial collateral ligament on the femoral side in order to widen the medial working space and prevent iatrogenic cartilage injury.

**FIGURE 4:**
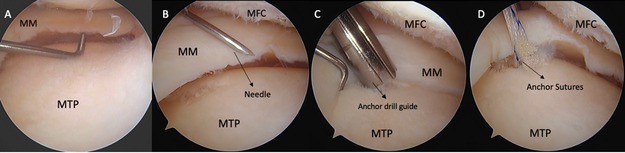
Arthroscopic views of the left knee showing the steps of the medial meniscus posterior root centralization. (A) An extruded medial meniscus detached from the medial tibial plateau. (B) Use of a needle, indicated by the arrow, to determine the optimal location for establishment of the accessory medial portal. (C) The anchor drill guide is positioned anterior to the medial meniscus and seated at the edge of the medial tibial plateau for anchor placement. (D) As indicated by the arrow, all‐suture anchor sutures after anchor insertion. (MFC, medial femoral condyle; MM, medial meniscus; MTP, medial tibial plateau.)

Using a probe, the degree of extrusion is assessed, and the desired amount of cartilage coverage is determined (Figure 4A). To increase meniscal mobility, the meniscotibial ligament is released using a soft‐tissue elevator. After simulating the intra‐articular placement and drilling trajectory of the anchor guide with a needle (Figure 4B), an accessory medial portal is created approximately 1 cm superior and 2 cm medial to the FM portal through a 5 mm incision. This portal serves as an entry point for the anchor to reach the medial border of the medial tibial plateau.

The drill guide is positioned precisely at the medial tibial margin, targeting the location that will allow centralization of the medial meniscus body (Figure 4C). Drilling is performed with a 2.7 mm drill, and a 3.0 mm all‐suture anchor (Seges, Ankara, Turkey) is inserted into the prepared socket (Figure 4D).

**FIGURE 5:**
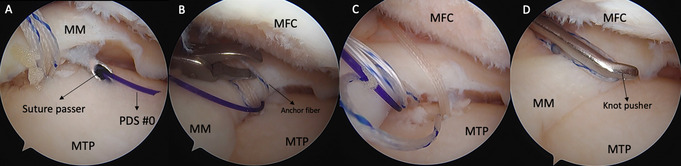
Arthroscopic views of the left knee showing suture shuttling and fixation during medial meniscus centralization. (A) A suture passer is entering through the meniscocapsular junction and delivering a No. 0 polydioxanone (PDS) shuttle suture beneath the medial meniscus into the joint. (B) Retrieval of the anchor fiber from outside the joint, together with the shuttle PDS suture, using a suture retriever. (C) Shuttling of the anchor fiber using the PDS shuttle suture. (D) Medial meniscus centralization achieved using a knot pusher with the paired fibers after completion of the shuttling process. (MFC, medial femoral condyle; MM, medial meniscus; MTP, medial tibial plateau; PDS, polydioxanone suture.)

With visualization from the anterolateral portal, a suture passer is used to shuttle 2 limbs of the all‐suture anchor through the meniscocapsular junction, spaced 5 mm apart, and the sutures are tied down via Revo knots to recenter the medial meniscus body (Figure 5).

### Medial Meniscus Posterior Root Repair

**FIGURE 6:**
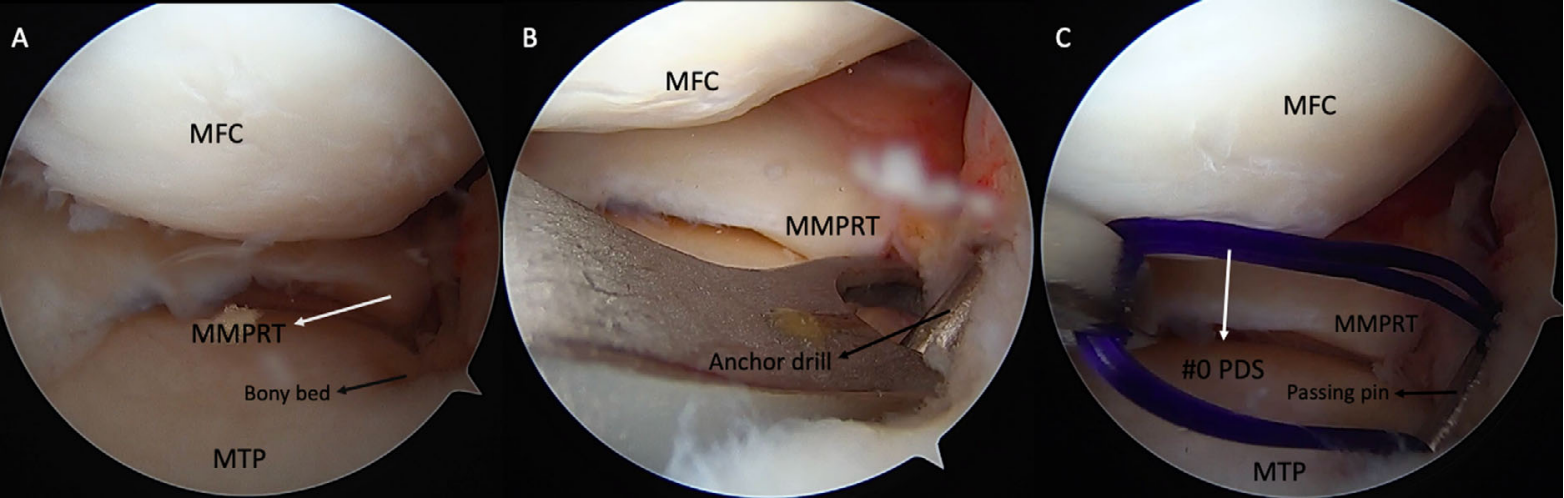
(A) Arthroscopic view showing the torn medial meniscus posterior root (white arrow) prior to repair and the prepared bony bed for root fixation (black arrow). (B) The anchor drill advanced through the meniscus posterior root repair guide and aligned over the prepared bony bed to create the tibial tunnel (black arrow). (C) A looped No. 0 PDS shuttle suture was passed through the created tibial tunnel and retrieved extra‐articularly (white arrow), loaded on a passing pin (black arrow). (MFC, medial femoral condyle; MMPRT, medial meniscus posterior root tear; MTP, medial tibial plateau; PDS, polydioxanone suture.)

The root tear is confirmed using a probe, and its reducibility is assessed with an arthroscopic grasper. To achieve a stable reduction of the posterior root, tear edges and surrounding scar tissue are refreshed using an arthroscopic shaver. To promote optimal bone‐meniscus integration and enhance biological healing, the cartilage surface at the planned reduction site is gently debrided using a curved arthroscopic curette to prepare an appropriate bony bed (Figure 6A).

A 1 cm skin incision is made between the medial border of the tibial tubercle and the deep medial collateral ligament. After blunt dissection, the meniscus root repair guide (Arthrex, Naples, FL) is set to 55° and inserted into the joint through the FM portal. The intraarticular component of the guide is positioned at the center of the prepared bony bed on the tibial plateau (Figure 6B), while the extraarticular component is positioned toward the anteromedial tibial surface. A 2.7 mm drill is used to create an antegrade tibial tunnel. After visualizing the drill bit entering the joint, the guide is held firmly in position, and the drill is removed.

A No.0 PDS loop is prepared and loaded onto a 2.4 mm × 430 mm passing pin such that the loop protrudes 2 to 4 mm beyond the pin tip. The passing pin is advanced through the guide and the tunnel until the loop reaches the joint surface. Once the pin exits intraarticularly, the guide is released and removed. Using a suture retriever through the FM portal, the PDS loop is retrieved from the joint (Figure 6C), and the passing pin is withdrawn.

**FIGURE 7:**
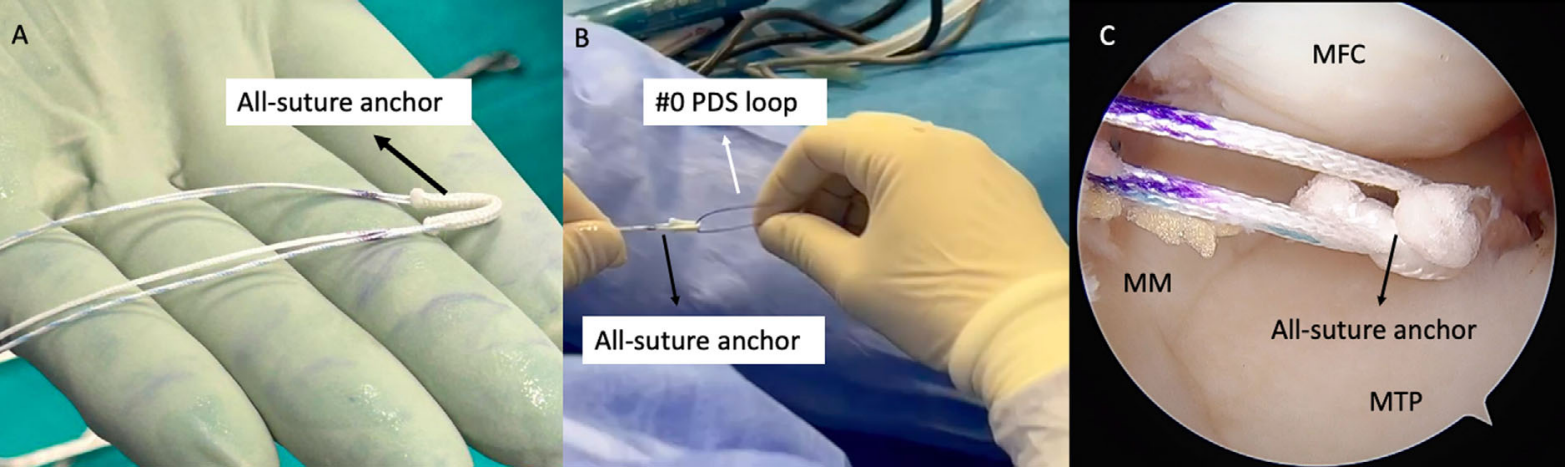
Extra‐articular and arthroscopic views showing preparation and implantation of the all‐suture anchor during medial meniscus posterior root repair. (A) Extra‐articular view showing an all‐suture anchor removed from its driver and ready for implantation into the tibial tunnel. (B) Extra‐articular view showing the all‐suture anchor centered within the No. 0 PDS loop to facilitate transfer of the anchor into the joint. (C) Arthroscopic view showing implantation of the all‐suture anchor into the prepared tibial tunnel. (MFC, medial femoral condyle; MM, medial meniscus; MTP, medial tibial plateau; PDS, polydioxanone suture.)

Outside the joint, a 3.0 mm all‐suture anchor (Figure 7A) (Seges, Ankara, Turkey) is detached from its screwdriver and placed into the PDS loop (Figure 7B). Pulling the free ends of the PDS from the tunnel orifice on the anteromedial tibial surface shuttles the all‐suture anchor retrograde through the tibial tunnel from the FM portal (Figure 7C). After confirming rigid anchorage within the tunnel, the anchor sutures are retrieved through the anterolateral portal using a suture retriever. Then, a silicone cannula (Arthrex, Naples, FL) is inserted into the FM portal.

**FIGURE 8:**
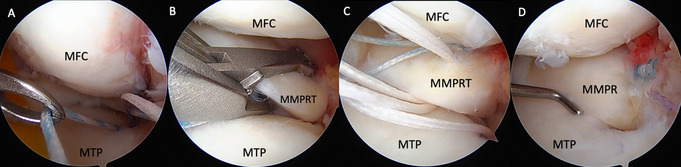
Arthroscopic views of the left knee showing passage of the suture limbs through the medial meniscus posterior root to achieve repair. (A‐C) Paired sutures of the implanted all‐suture anchor being grasped with a suture retriever and sequentially passed through the medial meniscus posterior root using a suture passer. (D) Arthroscopic view showing the tied knots and the final appearance of the repaired medial meniscus posterior root tear following fixation. (MFC, medial femoral condyle; MMPRT, meniscus posterior root tear; MTP, medial tibial plateau.)

**FIGURE 9:**
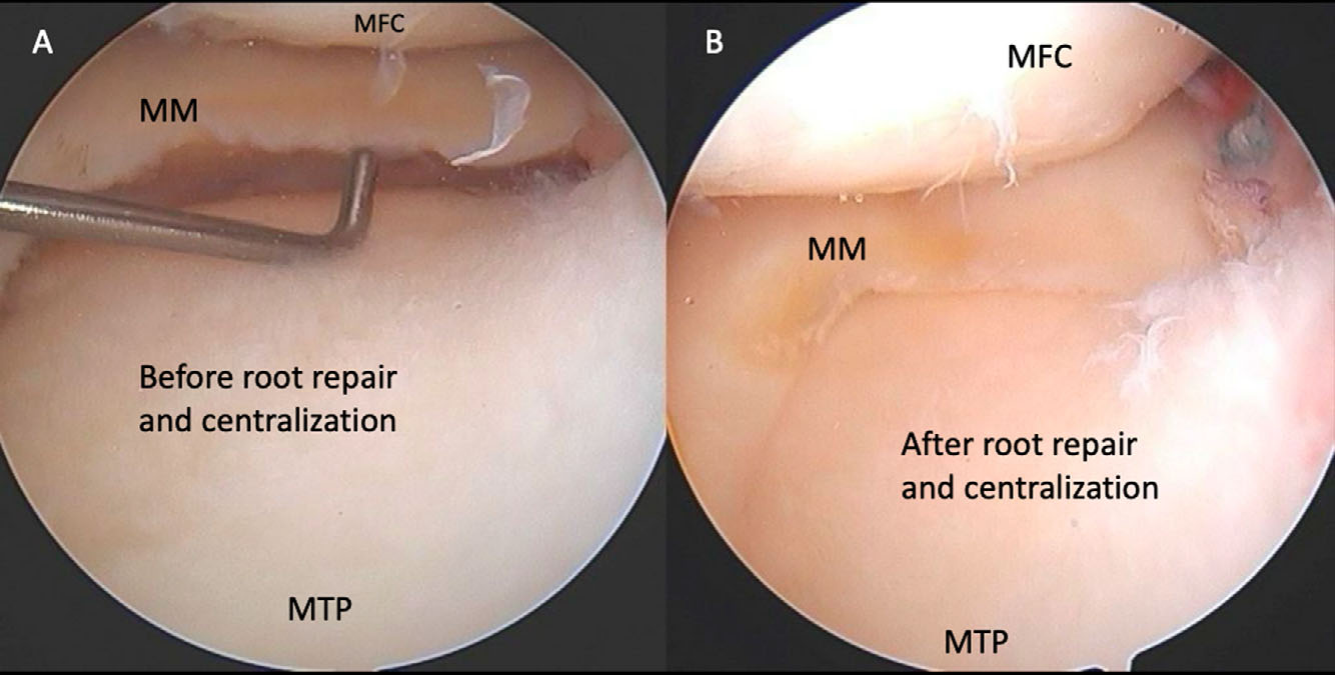
Arthroscopic views of the left knee showing the effect of medial meniscus posterior root repair combined with centralization. (A) Arthroscopic view showing the medial meniscus before posterior root repair and centralization, with persistent extrusion from the medial tibial plateau. (B) Arthroscopic view showing the medial meniscus after posterior root repair and centralization, showing restoration of the meniscus over the medial tibial plateau. (MFC, medial femoral condyle; MM, medial meniscus; MTP, medial tibial plateau.)

Next, the first pair of anchor sutures designated for passage through the root is brought into the cannulated FM portal. The sutures are sequentially passed through the medial meniscus posterior root by using a Mini Scorpion suture passer (Arthrex, Naples, FL). To increase the footprint contact area of the root, only one limb of the second suture pair is passed through the root. The suture pairs are then tied using the Revo knot technique, ensuring that the knots are positioned on the superior surface of the meniscus and adequate tension is applied to achieve rigid fixation. (Figure 8). The pre‐ and postoperative arthroscopic images of the medial meniscus are shown in Figure 9. The advantages and disadvantages of the procedure are listed in Table 4.

**TABLE 4:** Advantages and Disadvantages of This Procedure

**Advantages**	**Disadvantages**
Avoids the need for a posteromedial portal	Fixation strength may be affected by bone quality
Reduced risk of iatrogenic cartilage damage	The absence of a posteromedial portal may initially restrict visualization and instrument maneuverability, particularly early in the learning curve
Reduced risk of neurovascular damage	Potential risk of meniscal over‐constraint
Use of all‐suture anchors preserves bone stock	

### Postoperative Rehabilitation Protocol

Patients remain toe‐touch bearing for 4 weeks with an unloader knee brace locked in full extension. Attention is given to regaining full extension of the knee. In the first 2 weeks, knee flexion greater than 90° is not permitted. After 2 weeks, the range of motion is advanced as tolerated. After 4 weeks, progressive weight‐bearing starts. Squats greater than 90° are avoided for 6 months. Return to noncontact sports is permitted approximately 9 months postoperatively.

## DISCUSSION

This technical note offers the advantages of both the transtibial pull‐out technique and conventional suture anchor repair.

Retrograde tunnel creation facilitates enhanced diffusion of biological healing factors into the joint. An advantage compared with the transtibial pull‐out technique is that suture fixation to the anteromedial tibial cortex using an endobutton or double‐button devices is unnecessary. Instead, the sutures are tied directly onto the meniscal root with adjustable tension, preventing microlaxity along the repair construct, commonly described in the literature as the “bungee effect.”

According to a study by Cinque et al.,^15^ conventional suture anchor repairs showed reduced medial meniscal extrusion and lower medial compartment contact pressures after cyclic loading compared with the transtibial pull‐out technique. These biomechanical advantages may contribute to superior long‐term outcomes in patients undergoing anchor‐based meniscal root repair. Similarly, in a porcine knee model, Saengpetch et al.^16^ reported that both conventional suture anchors and transtibial all‐suture anchors were more effective than the transtibial pull‐out method in reducing extrusion and resisting axial loading.

The notable distinction between this technical note and conventional suture anchor techniques is that the antegrade creation of a tibial tunnel eliminates the need for an additional posteromedial portal. We believe that this modification prevents potential difficulties and risks associated with posteromedial anchor placement.

Previous clinical studies have shown that adding a centralization procedure to root repair effectively reduces meniscal extrusion.^17^ The goal of the centralization is not only to reduce postoperative extrusion but also to recenter the medial meniscus on the tibial plateau, thereby decreasing cartilage contact forces and reducing traction stress on the repaired posterior root.^18^ In this technical note, centralization is performed via all‐suture anchors used to pass sutures through the meniscocapsular junction and tie down the meniscal border to the medial margin of the tibial plateau.

There is also an emerging trend toward combining medial meniscus root repair with concurrent high tibial osteotomy.^19^ As the anchor described in this technique is positioned proximal to the typical osteotomy line, potential interference between the osteotomy and root repair is avoided in patients requiring concomitant high tibial osteotomy.

Despite the potential advantages of this combined arthroscopic technique, several limitations should be acknowledged. The fixation strength of all‐suture anchors may be affected by bone quality, particularly in osteoporotic tibial bone. In addition, the absence of a posteromedial portal may limit visualization and instrument maneuverability.

In conclusion, we believe that this technique offers a robust approach for reducing meniscal extrusion and minimizing stress on the repaired medial meniscus posterior root.

## DISCLOSURES

The authors (C.G., B.M.A., I.F., P.A., F.B., H.A.A.) declare that they have no known competing financial interests or personal relationships that could have appeared to influence the work reported in this paper.
